# Native Knee Septic Arthritis Due to Cutibacterium acnes: A Case Report

**DOI:** 10.7759/cureus.33183

**Published:** 2022-12-31

**Authors:** André Ferreira dos Santos, David V Peres, Cecília Barros, Pedro Varanda, Filipe Carriço

**Affiliations:** 1 Orthopaedics and Traumatology, Centro Hospitalar do Baixo Vouga, Aveiro, PRT; 2 Infection and Antibiotic Resistance Prevention and Control Unit, Hospital Pedro Hispano, Matosinhos, PRT; 3 Orthopaedics and Traumatology, Hospital de Braga, Braga, PRT

**Keywords:** native knee joint, gram-positive bacillus, cutibacterium acnes, uncommon pathogen agent, septic arthritis

## Abstract

Commensal skin anaerobes have been described as causative agents of prosthetic joint infections. Infection of native joints by these agents are, however, less common. We present the case of a 34-year-old male with recurrent joint effusion following closed trauma to the knee, four years ago, refractory to corticosteroid injections and several arthrocenteses. A synovial biopsy revealed *Cutibacterium acnes* infection leading to antibiotic therapy with clindamycin, and the patient was referred to orthopaedic and submitted to arthroscopic lavage. Atypical cartilage lesions, resembling the “growth of bacterial colonies”, were found in the tibial plateaus with repeated isolation of *C. acnes*. Inpatient treatment with penicillin and vancomycin was conducted, followed by an oral course of amoxicillin, with no further registered recurrences. In this case, the authors describe a rare cause of native knee septic arthritis while highlighting the importance of repeated microbiology studies and adequate collection technique and sample handling, in order to better ascertain whether the isolated agent represents a contaminated sample or a true infection.

## Introduction

Human skin commensal flora comprises diverse microorganisms. Common anaerobes such as *Cutibacterium* (*Propionibacterium*) species have been highlighted in orthopaedic research [1,2]. The following case illustrates a rare native knee septic arthritis caused by *Cutibacterium acnes* (*C. acnes*). This gram-positive bacillus is identified as a common agent of prosthetic shoulder infection, as well as other orthopaedic devices [1-4]. The clinical course is indolent and lacks typical signs of infection [4-6]. The most frequent site of colonization of *C. acnes* is the shoulder, accounting for higher infection rates in the upper body [7]. Hip and knee arthroplasties are affected less commonly [3]. However, infection of native joints by this pathogen is still widely underdiagnosed in clinical practice. To our knowledge, there are a total of seven reported cases of native knee *C. acnes* septic arthritis [2,4].

## Case presentation

The patient is a 34-year-old healthy male presenting with a four-year history of right knee pain, warmth, and recurrent effusion, secondary to a closed joint injury after falling from a motorcycle at a standstill. No fever, erythema, open wound, or fistula were described. There was no personal or family history of rheumatological disease. He denied any constitutional symptoms and was systemically well.

In the first two years, transient remission of symptoms was observed after treatment with, at least, three corticosteroid injections and 10 arthrocenteses. In October 2019, after a Magnetic Resonance Imaging (MRI) describing unspecific synovitis and rupture of the internal meniscus, a knee arthroscopy with synovectomy and a partial meniscectomy of both meniscus was performed. Two further joint aspirations and one corticosteroid injection were needed, without sustained improvement. In February 2021, a synovial biopsy was performed by rheumatology with the identification of *C. acnes* and *Atopobium vaginae.* Antibiotic therapy was started with clindamycin 600 mg every eight hours for three weeks, without clinical improvement. Microbiology cultures of the last two arthrocenteses and the one collected through the biopsy were negative. No crystals were observed and the pathology revealed no pathological findings.

**Figure 1:**
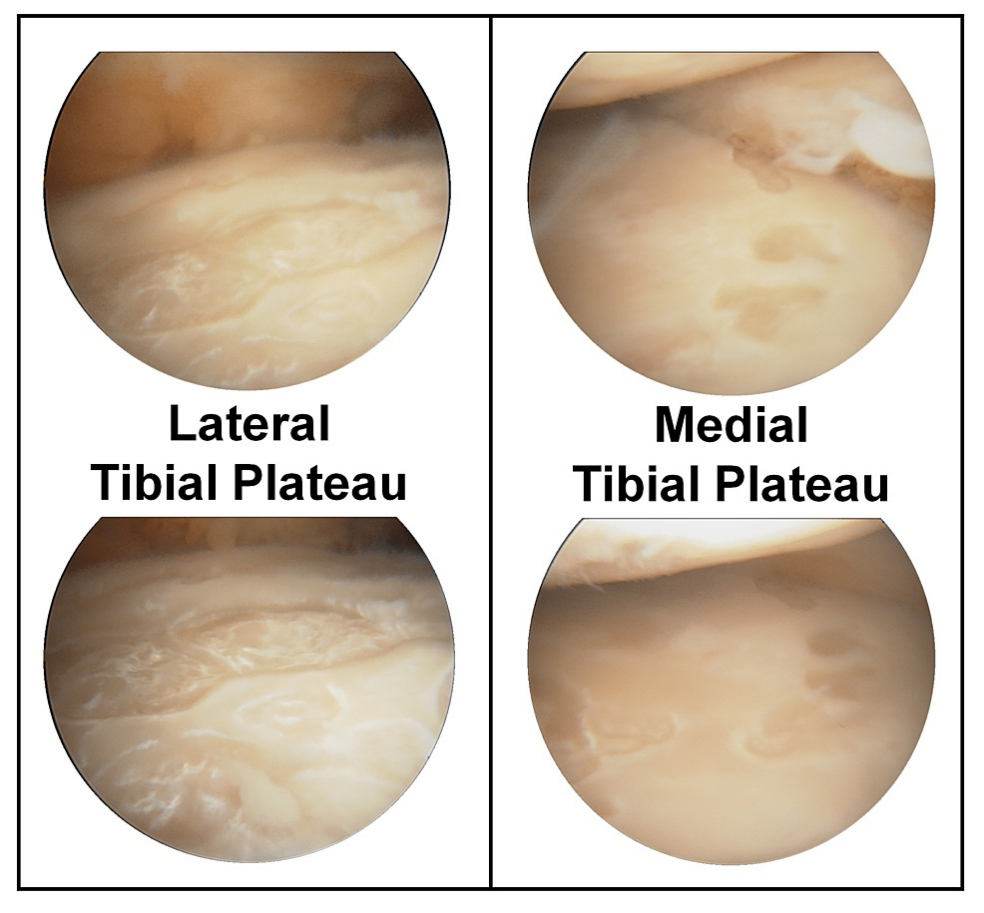
Right knee arthroscopy revealing atypical cartilage lesions, resembling the “growth of bacterial colonies”, found at the tibial plateaus.

Two months later, the patient was referred to orthopaedics and submitted to arthroscopic lavage. Atypical cartilage lesions, resembling the “growth of bacterial colonies”, were identified in the tibial plateaus (Figure 1). Biopsy sample identified, once more, *C. acnes*, however, aspirate culture was negative.

Patient was admitted for antibiotic treatment: two weeks of vancomycin 1000 mg every 12 hours, following one week of penicillin, three million units every four hours (Penicillin G sodium alternating with Penicillin G potassium). After discharge, oral amoxicillin 500 mg every eight hours was prescribed until the completion of a six-week course of antibiotic treatment. The length of six weeks of antibiotic treatment was decided based on the previous period of antibiotic with clindamycin, the difficulty to get therapeutic levels of vancomycin during the first week, and the prolonged and atypical presentation. At eight weeks of follow-up, the patient presented no symptoms or knee effusion.

## Discussion

Considering that *C. acnes* is a commensal microorganism with low pathogenicity, identification as an etiologic agent may be challenging since its growth in microbiological cultures can be interpreted as contamination. It is recommended that cultures be incubated for at least 14 days [1,4,6,7] to avoid misinterpretation of results, together with an adequate collection technique and handling of microbiological samples to prevent contamination [1,6].

In this case, while the synovial biopsy sample could be interpreted as contamination, *C. acnes* was once again isolated in the surgical biopsy. Additionally, the indolent clinical course, presenting with local warmth and joint effusion, without the typical signs or symptoms of septic arthritis, supports this diagnosis [2,4,6].

Regarding treatment, it is important to highlight that the duration of the infection presents a greater risk of permanent cartilage damage [8-10]. At the arthroscopy performed in 2019, no cartilage lesions were described. The surgery itself, subsequent arthrocentesis, and corticosteroid injections could have inoculated *C. acnes*. However, the lesion number and size described in the last arthroscopy (Figure 1) suggest a prolonged pathological process. The macroscopic characteristics of the lesions remain, nonetheless, unclear. To our knowledge, there are no other cases with these lesions described in the literature.

Early surgical intervention and prolonged antimicrobial therapy are, therefore, challenged by the indolent course of *C. acnes* [2,4,6,8]. In this case, despite the prolonged clinical manifestations, it can be hypothesized that, by treating the infection, the pro-inflammatory cycle was interrupted.

## Conclusions

*C. acnes* is an uncommon cause of septic arthritis in native joints, especially in lower limb joints. Combined with diagnostic difficulties, it is important to ensure adequate management of microbiological samples so as to achieve early diagnosis and initiation of treatment, preventing further joint damage and other complications.
